# Anti-Swelling Dual-Network Zwitterionic Conductive Hydrogels for Flexible Human Activity Sensing

**DOI:** 10.3390/polym17162230

**Published:** 2025-08-16

**Authors:** Zexing Deng, Litong Shen, Qiwei Cheng, Ying Li, Qianqian Liu, Xin Zhao

**Affiliations:** 1 College of Materials Science and Engineering, Xi’an University of Science and Technology, Xi’an 710054, China24211225047@stu.xust.edu.cn (L.S.); chengqiweixust@163.com (Q.C.); liyingxust@xust.edu.cn (Y.L.); liuqq@xust.edu.cn (Q.L.); 2 State Key Laboratory for Mechanical Behavior of Materials, Xi’an Jiaotong University, Xi’an 710049, China

**Keywords:** zwitterionic hydrogels, dual-network, ionic conductivity, anti-swelling, flexible sensors, human motion monitoring

## Abstract

Conventional conductive hydrogels are susceptible to swelling in aquatic environments; which compromises their mechanical integrity; a limitation that poses a potential challenge to their long-term stability and application. In this study, a zwitterionic ion-conductive hydrogel was fabricated from polyvinyl alcohol (PVA), acrylic acid (AA), and [2-(methacryloyloxy)ethyl]dimethyl-(3-sulfopropyl)ammonium hydroxide (SMBA), forming a dual-network structure. A copolymer of zwitterionic SBMA and AA formed the first network, and PVA formed the second network by repeated freeze–thawing. The equilibrium state of zwitterionic SBMA was modulated by AA to protonate the SBMA, which resulted in the conversion of -SO_3_^−^ to -SO_3_H; thus, hydrogels had the anti-swelling property driven by electrostatic repulsion. In addition, the prepared hydrogels possessed excellent mechanical properties (tensile strength of 0.76 MPa, elongation at break of 322%, and compressive strength of 0.97 MPa at 75% compressive strain) and remarkable anti-swelling properties (80% swelling after 120 h of immersion). Owing to the zwitterionic nature of SBMA, the hydrogel also showed inherent antimicrobial properties and high electrical conductivity, which could be capable of monitoring human movement and physiological signals. This work provides a facile strategy for designing hydrogels with remarkable mechanical properties and anti-swelling characteristics, expanding the application environment of hydrogels in flexible sensing

## 1. Introduction

Conductive hydrogels [1,2,3] have emerged as promising candidates for utilization in flexible sensing [4,5], electronic skin [6,7,8], and soft robotics [9,10] because of their unique intrinsic flexibility and tunable electrical conductivity properties [11]. Nevertheless, owing to the existence of hydrophilic groups in the macromolecular chain, conductive hydrogels can lead to significant volume expansion after absorbing a large amount of water in aquatic environments. The excessive volume expansion can negatively affect the mechanical properties and electrical conductivity of conductive hydrogels [12,13], which can significantly limit their practical utilization in flexible sensing [14,15].

To overcome this limitation, several anti-swelling strategies have been developed, including electrostatic repulsion [16,17], multiple crosslinking mechanisms [18,19,20,21], or introduction of hydrophobic components [22,23]. For instance, PVA and amylopectin (AMY) could form a dense network through firm hydrogen bonding, and this could be followed up with the salting-out method to prepare highly tough, anti-swelling ionic-conductive hydrogels [24]. Further, researchers developed a multifunctional anti-swelling conductive hydrogel incorporating chemically tailored carboxymethyl chitosan (CMCS) and tannic acid (TA) through multiple crosslinking [25]. These advancements highlight the great potential of anti-swelling conductive hydrogels in broadening the scope of hydrogel-based devices. Notably, research integrating the aforementioned dual strategies to achieve anti-swelling conductive hydrogels remains relatively unexplored.

Zwitterionic polymers possess balanced cationic and anionic groups with uniform charge distribution along polymer chains, resulting in overall electrical neutrality. The coulombic interactions between oppositely charged moieties in these zwitterionic polymers, coupled with electrostatic interactions between charged groups and electrolyte ions, can significantly enhance ionic signal transmission [26]. [2-(Methacryloyloxy)ethyl]dimethyl-(3-sulfopropyl)ammonium hydroxide (SMBA), as a zwitterionic monomer, exhibits pH-responsive characteristics [27]. PolySBMA is able to transfer from a neutral polymer to a positively charged polymer through adjusting the pH value, which regulates the osmotic pressure of the PolySBMA-based hydrogel through electrostatic interactions, thus making the hydrogel with anti-swelling property [28]. Furthermore, poly(vinyl alcohol) (PVA), as a non-toxic polymer, has been extensively employed as a matrix for a high-strength hydrogel. The abundant hydroxyl groups on PVA chains facilitate the formation of inter- and intramolecular hydrogen bonds, while also enabling reactions with diverse functional groups to form chemically or physically crosslinked hydrogel networks [29,30].

In this work, we developed an anti-swelling ionic-conductive hydrogel with dual-network structure by using zwitterionic monomer SBMA and AA through copolymerization as the first network. PVA formed the second network by repeated freeze–thawing. The adjustment of the equilibrium state of zwitterionic ions by AA protonates the SBMA monomer and reduces the osmotic pressure of the hydrogel, which yields excellent anti-swelling properties, reaching 80% swelling ratio upon 120 h water immersion. Furthermore, this approach elevated the hydrogel’s mechanical performance, demonstrating 0.76 MPa tensile strength, 322% elongation at break, along with 0.97 MPa compressive strength at 75% strain. The hydrogel exhibited good sensitivity (gauge factor GF = 3.75) in the tensile strain range of 150–250%, and showed rapid response and recovery characteristics (269 ms and 87 ms, respectively). In addition, the prepared hydrogel exhibited desirable antibacterial activity and could monitor human motion and physiological signals in both air and aqueous environments, broadening hydrogel utilization in flexible sensing applications.

## 2. Experimental Section

Materials: [2-(methacryloyloxy)ethyl]dimethyl-(3-sulphonatopropyl)ammonium hydroxide (SBMA), acrylic acid (AA), polyvinyl alcohol 1799 (PVA, Mn = 75,000, ≈98–99% alcoholysis), and phosphate buffer (PBS, pH 7.4) were provided by Aladdin (Shanghai, China). Ammonium persulfate (APS) and N, N’-methylenebisacrylamide (BIS) were obtained from Sigma-Aldrich (Shanghai, China). All reagents and solvents were analytically pure.

Preparation of Hydrogel: First, 1.0 g of PVA was completely dissolved in 7 mL of deionized (DI) water at 90 °C (2 h) with completely sealed to avoid water evaporation. After cooling to 50 °C, a mixture of 2 mL AA and 1.2 g SBMA dissolved in 2 mL DI water was added to PVA solution, forming a homogeneous solution. Subsequently, 300 μL of APS (100 mg/mL) and 5 mg of BIS were added to the previous solution, followed by rigorous stirring for 5 min. The precursor hydrogel solution was subsequently poured into a mold and polymerized at 70 °C. After polymerization, the crosslinked hydrogels underwent a freeze–thaw cycle for 3 times (−20 °C for 3 h, then 25 °C for 3 h) to yield a PVA/AA1-SBMA hydrogel. PVA/SBMA, PVA/AA2-SBMA, and PVA/AA3-SBMA hydrogels were prepared identically. The content of hydrogel for each component is shown in the following Table 1.

Characterization of hydrogels: Chemical structures of lyophilized hydrogels were analyzed by Fourier transformed infrared spectroscopy (FT-IR; PerkinElmer Spectrum Two, Waltham, MA, USA) and X-ray differentiation (XRD; Bruker D8 Advance, Billerica, MA, USA) measurements. The physical structure of hydrogels before and after swelling was examined using a scanning electron microscope (SEM; Hitachi S4800, Tokyo, Japan).

Mechanical properties: The mechanical performance of hydrogels was assessed with a universal testing machine. Hydrogel tensile specimens (25 mm of length × 4 mm of width × 3 mm of height) were tested with a tensile speed of 10 mm/min. Cyclic tensile testing was set as 100% of tensile strain with a speed of 50 mm/min. For compression, cylindrical hydrogel specimens (50 mm of diameter × 30 mm of height) were tested with a compressive speed of 80 mm/min. Cyclic compressive testing was set as 80% of compressive strain with a speed of 80 mm/min. Triplicate measurements were adapted to ensure reproducibility.

Swelling behavior: The hydrogel sample was weighed to obtain the initial mass (W_0_) and then immersed in DI water to allow swelling. At predefined time points, the sample was taken out, and the surface moisture was removed before measuring the swollen mass (W_s_). The swelling ratio (SR) was calculated the following equation [31]:

$$\mathrm{SR}=(W_{S}{-W}_{0})/W_{0}*100\%$$

Antibacterial activities: *S. aureus* (Gram-positive) and *E. coli* (Gram-negative) were employed to assess the hydrogel’s antibacterial performance. Firstly, a 0.1 mL of aliquot of bacterial (*S. aureus* or *E. coli*) suspension with 1 of optical density value was added to 0.2 mL of phosphate-buffered saline (PBS) (blank control group) and 0.2 mL of hydrogel samples (experimental group) in a 24-well plate, followed by incubation at 37 °C for 1 h. After incubation, the bacterial suspension in each group was diluted with 0.9 mL of PBS, and 0.1 mL of bacteria diluted suspension was transferred to plates containing agar for cultivation of 24 h. The antimicrobial activity of the hydrogel was evaluated by enumerating colony-forming units (CFUs).

Cytocompatibility: To evaluate the cytotoxicity of hydrogels, a direct contact method was adapted. Before all, mouse fibroblast L929 cells (gifted from Chinese Academy of Sciences) were seeded in 96-well plates and cultured in Dulbecco’s modified eagle medium (DMEM) medium with 10% newborn calf serum (NBCS; Gibco, Thermo Fisher Scientific (Waltham, MA, USA)) at 37 °C with 5% CO_2_ for 24 h. Following medium removal and phosphate-buffered saline (PBS) rinsing, the cells were co-cultured with hydrogel samples (experimental group), while the control group remained untreated, and the medium was refreshed every 2 days. The following protocols could be found in our previous publication [32].

Electrical and sensing properties: The hydrogels were fabricated into discs with a thickness of 5 mm and a diameter of 8 mm. Electrical resistance measurements were performed on the hydrogel using a digital multimeter.

The conductivity (σ) of the sample was evaluated primarily through resistivity (ρ), the following formula was applied to calculate the resistivity [33]:

ρ=RS/L

where R is the resistance, L is the length of the hydrogel, and S is size of the cross-sectional area.

The conductivity was calculated by the following equation [34]:

σ=1/ρ

To monitor human movement, the hydrogel was first mounted on the skin of the target location. The copper wires were then connected with the hydrogel, followed secured with waterproof adhesive tape. To prevent water evaporation during testing, the hydrogel surface was coated with silicone oil. The hydrogel device was simultaneously connected to a digital multimeter for real-time signal acquisition. Resistance changes were determined during flexion movements of fingers, arms, and knee joints. The relative resistance change was calculated as [35]:

$$∆R/R_{o}=(R-R_{0})/R_{0}*100\%$$

where R_0_ and R correspond to the initial resistance and real-time resistance during deformation, respectively. Gauge factor (GF), an indicator of strain sensitivity, was determined by [36]:

$$GF=(\Delta R/R_{0})/\varepsilon$$

where ε denotes the applied strain.

## 3. Results and Discussion

### 3.1. Preparation of Hydrogels

**Scheme 1:**
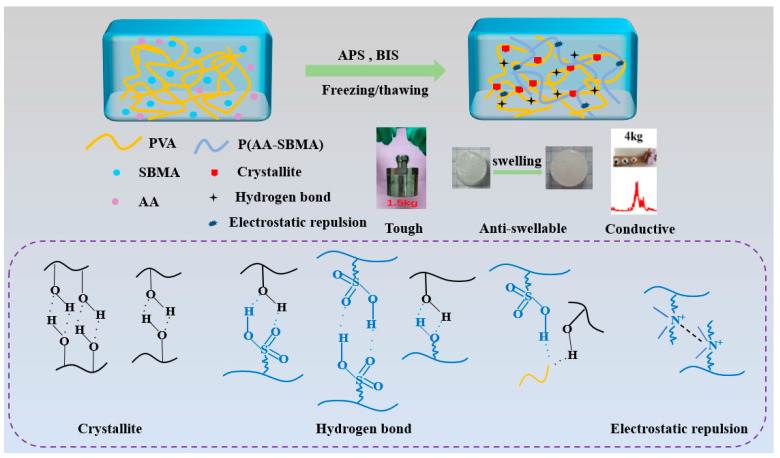
Preparation process and formation mechanism of the PVA/AA-SBMA hydrogel.

As shown in Scheme 1, PVA, AA and SBMA were firstly dissolved to induce protonation of SBMA. APS and BIS were then added to the above solution to initiate polymerization and crosslinking, respectively, forming the first network composed of a zwitterionic SBMA–AA copolymer. We followed constructed a second PVA network through cyclic freeze–thawing. Due to the acidic environment provided by AA, the -SO_3_^−^ in SBMA monomers was converted to -SO_3_H. This protonation process not only significantly enhanced the mechanical properties through increased hydrogen bonding [37], but also created electrostatic repulsion between inherent quaternary ammonium groups, effectively suppressing water molecules penetration within the hydrogel network and consequently reducing the swelling ratio of hydrogels. For comparative studies, PVA/SBMA hydrogel without AA was also prepared.

### 3.2. Characterization of Hydrogels

**Figure 1:**
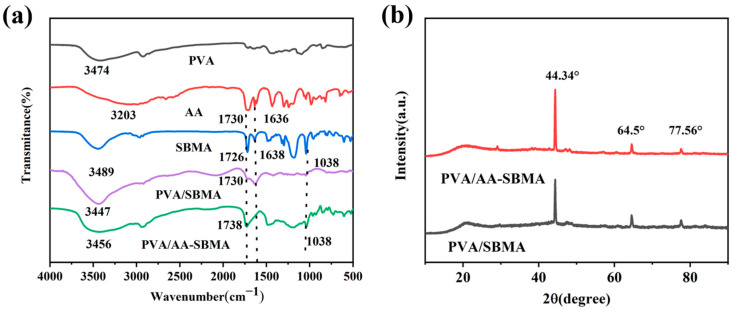
(**a**) FT-IR spectra of PVA, AA, SBMA, PVA/SBMA and PVA/AA-SBMA. (**b**) XRD spectra of PVA/SBMA and PVA/AA-SBMA.

To elucidate the chemical structure of PVA/AA-SBMA hydrogels, we performed comprehensive FT-IR and XRD analyses. As denoted in Figure 1a, FT-IR spectroscopy revealed characteristic peaks at 3474 cm^−1^ and 3489 cm^−1^ corresponding to the hydroxyl (-OH) group stretching vibrations of PVA and SBMA [38,39], respectively. Notably, these -OH stretching vibrations shifted to lower wavenumbers (3447 cm^−1^ for PVA/SBMA and 3456 cm^−1^ for PVA/AA-SBMA), demonstrating enhanced hydrogen bonding within the hydrogel networks [40]. Moreover, the characteristic sulfonic acid groups peak at 1038 cm^−1^ exhibited substantial attenuation in PVA/AA-SBMA hydrogels compared to pure SBMA, confirming the protonation of zwitterionic SBMA under acidic conditions. In addition, characteristic peaks of carbon–carbon double bonds in monomer AA (1636 cm^−1^) and SBMA (1638 cm^−1^) disappeared in the PVA/AA-SBMA curve, indicating the polymerization of AA and SBMA.

XRD analysis (Figure 1b) revealed distinct diffraction peaks at 2θ = 44.34°, 64.5°, and 77.56° for both PVA/SBMA and PVA/AA-SBMA hydrogels. These characteristic peaks demonstrate that the cyclic freeze–thaw process induced an ordered arrangement of PVA molecular chains [41], while simultaneously enhanced intermolecular interactions. This crystalline reorganization provided the thermodynamic driving force for microcrystalline domain formation within the hydrogel networks [42].

**Figure 2:**
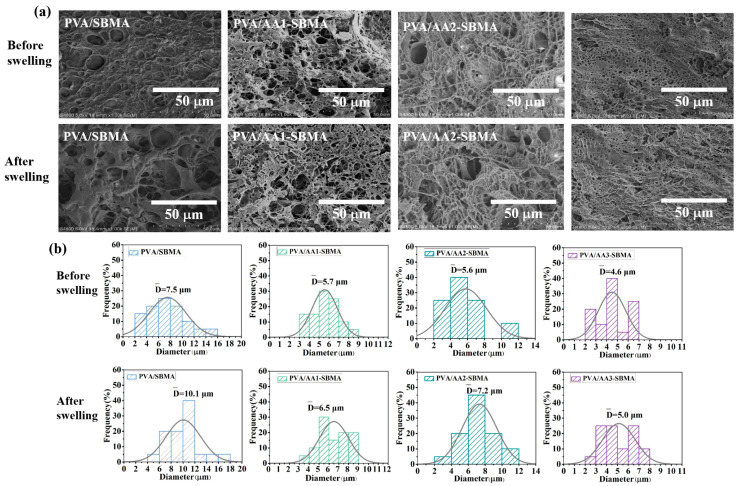
PVA/SBMA and PVA/AA-SBMA hydrogels before and after swelling. (**a**) SEM and (**b**) pore size distribution histograms.

SEM measurements were performed to systematically investigate the influence of AA on the micromorphology of hydrogels (Figure 2a). Comparative pictures of freeze-dried cross-sections revealed that both PVA/SBMA and PVA/AA-SBMA hydrogels exhibited porous structure. In addition, the porous structures of hydrogels were also documented after immersion in DI water. Notably, the PVA/SBMA hydrogel demonstrated substantial expansion of pore size after immersion in DI water for 120 h, while the PVA/AA-SBMA hydrogel maintained its original micromorphology without significant changes. This might because AA induced the protonation of sulfonic acid groups and the consequent enhancement of hydrogen bonding interactions [43]. Furthermore, the denser pores and network architectures were observed after the AA content increased from 2 mL to 4 mL before swelling and after swelling, indicating enhanced anti-swelling performance.

The structure evolution of hydrogels before and after swelling was further verified by the statistics of the pore size distribution of the hydrogels. As presented in Figure 2b, the results showed that PVA/SBMA hydrogels without AA addition exhibited a substantial increase in pore size after swelling 12 h. In contrast, when the AA content was increased to 2 mL and 4 mL, the pore sizes remained virtually unchanged before and after swelling. The average pore size of the PVA/SBMA sample was increased from 7.5 μm to 10.1 μm after swelling, while the average pore size of PVA/AA1-SBMA, PVA/AA2-SBMA, and PVA/AA3-SBMA samples was increased from 5.7 to 6.5 μm, 5.6 to 7.2 μm, and 4.6 to 5.0 μm, respectively. The PVA/SBMA sample displayed distinguish pore size increase compared with PVA/AA-SBMA samples. These results are beneficial for the anti-swelling performance of PVA/AA-SBMA hydrogels.

### 3.3. The Mechanical Properties of Hydrogels

**Figure 3:**
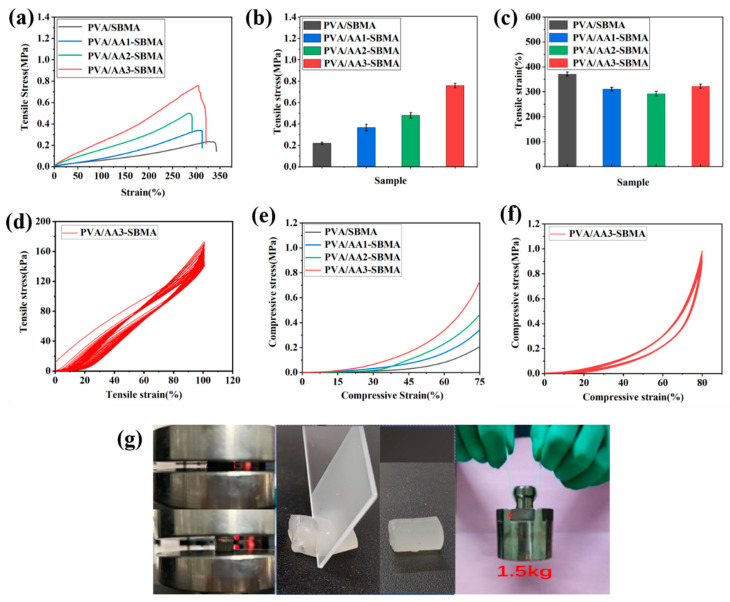
Mechanical properties of the PVA/AA-SBMA hydrogel. (**a**) Tensile stress–strain curves. (**b**) Elongation at break. (**c**) Tensile strength at break. (**d**) Tensile stress–strain curves of the PVA/AA3-SBMA hydrogel sample with 100% of strain for 20 cycles. (**e**) Compressive stress–strain curves. (**f**) Compressive stress–strain curves of the PVA/AA3-SBMA hydrogel sample with 80% of strain for 20 cycles. (**g**) Pictures presenting the mechanical properties of the PVA/AA-SBMA hydrogel.

A comprehensive investigation of the mechanical properties of PVA/AA-SBMA hydrogels with varying AA content was conducted through tensile stress–strain measurements (Figure 3a–c). The prepared hydrogel without AA addition exhibited relatively poor mechanical performance, demonstrating a tensile breaking stress of 0.22 MPa and tensile breaking strain of 371%. Upon incorporation of 2 mL or 3 mL of AA, the hydrogels showed significant improvement in tensile stress at break, reaching 0.37 MPa and 0.48 MPa, respectively, while experiencing a minor reduction in tensile strain to 311% and 292% due to the increasing crosslinking density of hydrogels. The optimal mechanical performance of hydrogel was achieved with 4 mL of AA content, where the hydrogel displayed a tensile breaking stress of 0.76 MPa coupled with a tensile breaking strain of 322%. The excellent mechanical properties resulted from two synergistic mechanisms. The freeze–thaw cycles facilitated the formation of hydrogen-bonded microcrystalline domains among PVA molecules, which simultaneously increased the crosslinking density and improved the tensile strength. Concurrently, the introduction of AA promoted protonation of the -SO_3_^−^ groups in SBMA to form -SO_3_H, generating supplementary intermolecular hydrogen bonds that further reinforced the mechanical performance of the hydrogel.

Cyclic tensile testing was also performed using the 4 mL of AA hydrogel, Figure 3d revealed distinct mechanical hysteresis behavior at 100% strain during first cycle. The initial cycle displayed a pronounced hysteresis loop attributed to the reversible breaking of hydrogen bonds between PVA, AA, and SBMA components. Remarkably, after 20 stretching cycles, the curves of the hysteresis loop exhibited significant stability without obvious difference, indicating the a highly stable network structure with exceptional fatigue resistance for our hydrogel.

As presented in Figure 3e, the mechanical compressive properties of hydrogels with varying AA contents were also comprehensively investigated. These results demonstrated excellent consistency with the tensile stress–strain measurements. The PVA/AA3-SBMA hydrogel exhibiting the highest compressive stress of 0.97 MPa at 75% strain. Further, cyclic loading–unloading tests were also performed, and revealed a nearly negligible hysteresis when the compressive strain was 80% for 20 cycles (Figure 3f). This remarkable compressive-recovery performance clearly demonstrated that the synergistic combination of double-network structure, microcrystalline domain, and hydrogen bonding conferred outstanding mechanical performance to the hydrogel. These findings suggest that the dual-network, microcrystalline domains of PVA, and multiple hydrogen bonds of hydrogel are beneficial for effective energy dissipation, ultimately providing the hydrogel with exceptional anti-fatigue performance [44].

The mechanical properties of PVA/AA-SBMA hydrogels were further verified through presentation pictures. As shown in Figure 3g, Movie S1, and Movie S2, these hydrogels demonstrated exceptional strength and toughness, exhibiting complete shape recovery after external stresses of compression or cutting removal. Furthermore, the hydrogels displayed remarkable load-bearing capacity, successfully supporting a 1.5 kg of weight without structure failure. These pictures provide supportive evidence for the superior mechanical performance of PVA/AA-SBMA hydrogels.

### 3.4. The Anti-Swelling Properties of Hydrogels

**Figure 4:**
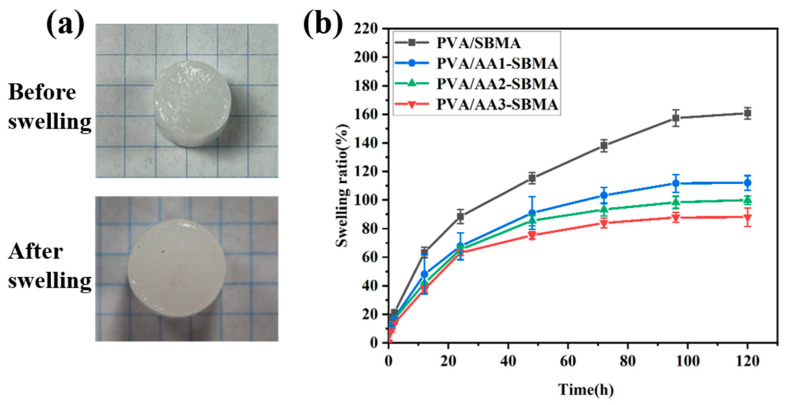
(**a**) Images of PVA/AA3-SBMA hydrogel before and after swelling. (**b**) Swelling ratio curves of hydrogels.

The long-term stability and performance of hydrogels in aquatic environments are often severely compromised by swelling effects. To address this critical issue, we systematically investigated the swelling behavior of PVA/SBMA and PVA/AA-SBMA hydrogels Visual observation revealed that PVA/AA-SBMA hydrogel exhibited limited swelling, showing only minor changes in shape and volume (Figure 4a). Quantitative swelling kinetics analysis through weight measurements (Figure 4b) demonstrated that PVA/SBMA hydrogel showed continuous swelling during the initial 96 h, reaching an equilibrium swelling ratio of approximately 160% after 120 h swelling. Importantly, the equilibrium swelling ratio of PVA/AA-SBMA hydrogels exhibited an obvious dependence on AA content. And the equilibrium swelling ratio would decrease progressively with increased concentration of AA. When the concentration of AA reached to 4 mL, the equilibrium swelling ratio of corresponding hydrogel was significantly reduced to 80%.

In order to compare the mechanical performance and electrical conductivity before and after swelling, we have conducted additional experiments and the corresponding results are shown in Figures S1 and S2. After swelling for one day, the electrical conductivity of the PVA/SBMA sample exhibited a substantial decrease while PVA/AA-SBMA samples maintain original levels without significant change (Figure S1). Similarly, the tensile strength of the PVA/SBMA sample also displayed an obvious decrease due to apparent swelling, which displayed structure fracture under minor tensile force (Figure S2a). For comparison, the PVA/AA3-SBMA sample still demonstrated good tensile strength that it can be stretched and recovered at an appropriate strain (Figure S2b). The PVA/AA3-SBMA samples demonstrated a small decrease in tensile strength (Figure S2c), further indicating the anti-swelling effect’s role in maintaining mechanical properties. This anti-swelling effect was originated from the acidic environment created by AA, which disrupted the zwitterionic equilibrium of SBMA. Specifically, the low pH conditions promoted protonation of -SO_3_^−^ groups, thereby generating electrostatic repulsion of the inherent positively charged quaternary ammonium groups (-R_3_N^+^). In addition, the double-network structure also promoted the anti-swelling properties of hydrogels. This intermolecular repulsion and double network effectively excluded water molecules enter the hydrogel network, thereby achieving good anti-swelling performance.

### 3.5. Antibacterial Activities of Hydrogels

**Figure 5:**
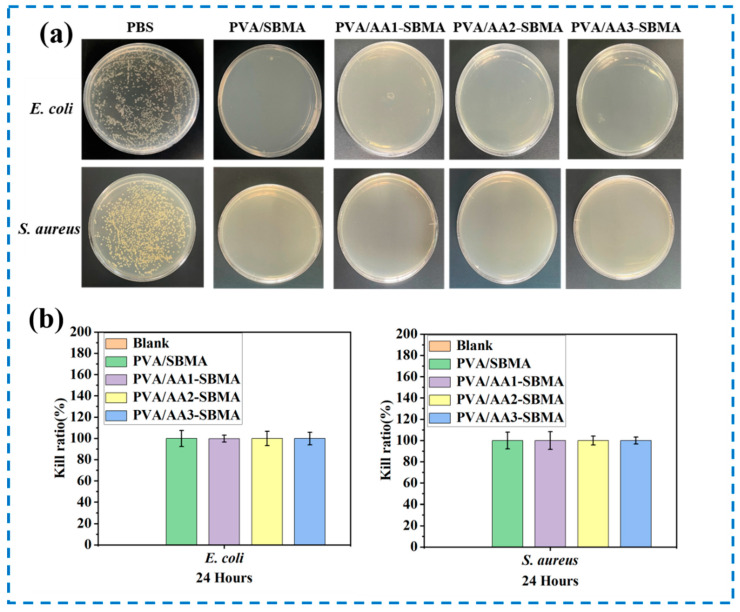
In vitro antibacterial activities of PVA/SBMA and PVA/AA-SBMA hydrogels. (**a**) Photographs of hydrogels against *E. coli* or *S. aureus*. (**b**) Antibacterial ratio against *E. coli* or *S. aureus*. Bars means the standard deviation of each group.

The antibacterial efficacy of the hydrogels was assessed via direct surface contact testing against both Gram-negative (*E. coli*) and Gram-positive (*S. aureus*) strains (Figure 5a). Firstly, hydrogel samples were incubated with *E. coli* or *S. aureus* for 1 h. Then, the bacterial suspension was diluted and transferred to agar plates for incubation. After 24 h of incubation, a striking contrast was noted between the control and experimental groups. While the control plates with only PBS exhibited dense bacterial colonization, all PVA/SBMA, PVA/AA1-SBMA PVA/AA2-SBMA, and PVA/AA3-SBMA hydrogel samples demonstrated remarkable antibacterial efficacy, with virtually no viable bacteria colonies detected on their culture plates. These results provide evidence for the broad-spectrum antibacterial activity of developed hydrogels.

A comprehensive quantitative evaluation of antibacterial activity was conducted through colony counting and subsequent antibacterial rate calculation (Figure 5b). The hydrogels exhibited remarkable bactericidal efficiency, achieving over 99% of elimination rates against both *E. coli* and *S. aureus*, thereby conclusively demonstrating their exceptional antibacterial capacity. The outstanding antibacterial performance stems from the synergistic action of dual mechanisms. The positively charged quaternary ammonium groups present in zwitterionic SBMA establish strong electrostatic interactions with negatively charged bacterial surfaces, resulting in substantial membrane disruption and consequent bacterial cell lysis [45]. Furthermore, the introduction of AA creates a weakly acidic microenvironment that not only inhibits bacterial growth but also promotes hydrolysis of critical surface components including proteins and nucleic acids, effectively preventing bacterial proliferation [46].

### 3.6. Biocompatibility of Hydrogels

**Figure 6:**
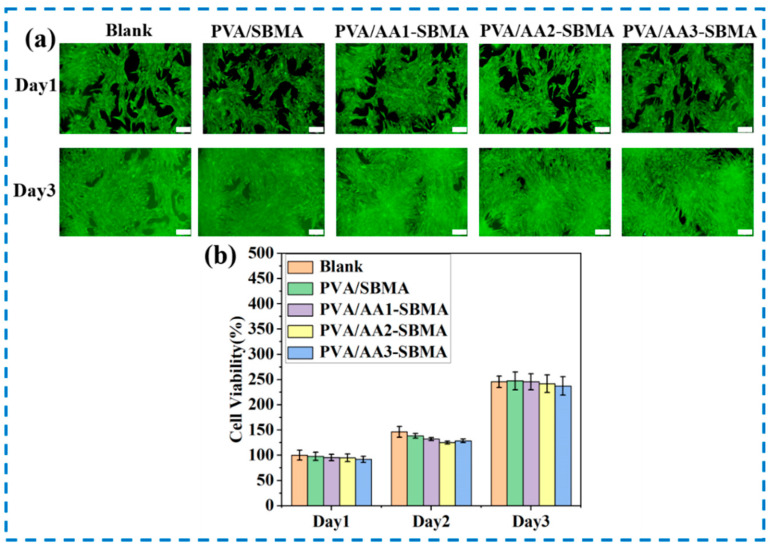
(**a**) Live/dead staining images of L929 cells after 1 and 3 days of co-cultivation. Scar bar = 200 μm. (**b**) Cell viability at different days. Bars means the standard deviation of each group.

The biocompatibility of PVA/SBMA and PVA/AA-SBMA hydrogels was evaluated through live/dead staining and CCK-8 assays (Figure 6). Hydrogel samples were rinsed with PBS before cocultured with cells to avoid potential toxicity. Live/dead staining results revealed that cells presented relatively dispersed within both PVA/SBMA and PVA/AA-SBMA on the first day of culture, extensive cell proliferation was observed on the third day, as evidenced by the widespread distribution of viable cells exhibiting characteristic spindle-shaped morphology. Notably, the absence of red-stained dead cells throughout the incubation period confirmed the non-cytotoxic nature of the hydrogels.

Complementary CCK-8 analysis demonstrated significant increase in cell viability from day 1 to day 2 and day 3 among all groups, with no statistically significant differences observed between hydrogel groups and control conditions. These collective findings provide evidence that both PVA/SBMA and PVA/AA-SBMA hydrogels possess excellent biocompatibility, supporting not only cellular survival but also proliferation without inducing cytotoxic effects.

### 3.7. The Sensing Properties of Hydrogels

**Figure 7:**
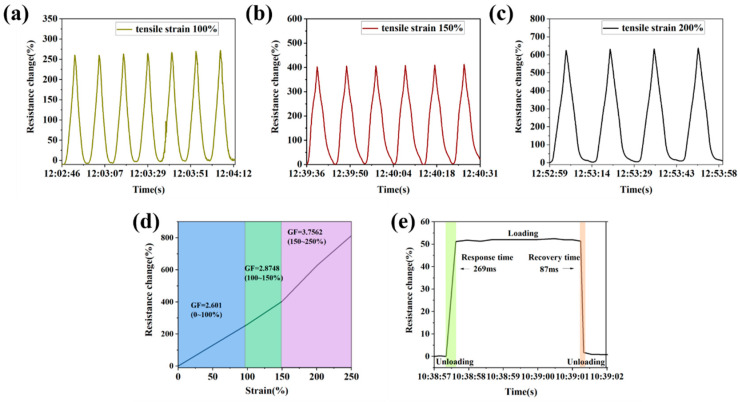
Relative resistance changes in PVA/AA3-SBMA hydrogel: (**a**) 100% strain, (**b**) 150% strain, and (**c**) 200% strain. (**d**) Sensitivity of PVA/AA3-SBMA hydrogel at different strain ranges. (**e**) Response and recovery times of the PVA/AA3- SBMA hydrogel.

The conductivity of hydrogels is shown in Figure S1. Before swelling, the corresponding conductivity range of hydrogels is 0.17–0.33 S/m. It can be found that the conductivity of hydrogels was decreased with the increased AA content due to the protonation of SBMA and increased crosslinking ratio, thereby less ions formed and conductive path constrained. After swelling, the conductivity of the PVA/SBMA sample demonstrated an obvious drop because significant swelling would lead to ions concentration diluted excessively. As a result, the low ions concentration limits the ability of ions to conduct even though with high mobility. On the contrary, PVA/AA-SBMA samples demonstrated increased conductivity because swelling would reduce ion migration resistance, and mobility is enhanced. Even with a slight dilution of ions concentration, the positive effects of ions mobility outweigh the negative effects of concentration dilution. Taking account of the mechanical performance, sensitivity, and anti-swelling performance of hydrogels, we selected the PVA/AA3-SBMA sample with the best mechanical performance and the lowest conductivity for strain sensing application (Figure 7a–c and Movie S3). The relative resistance change exhibited a strain-dependent response, showing a progressive increase from ~280% to ~630% of resistance changes when the tensile strain increased from 100% to 200%. During each stretch-recovery cycle, the resistance changes in hydrogel displayed stable variation, demonstrating stable sensing ability. This electromechanical behavior originated from the conductive pathways stretching during elongation, with subsequent recovery upon strain release. In addition, the hydrogel demonstrated linear sensitivity across different strain ranges, with a calculated GF value of 2.60 (0–100% strain), 2.87 (100–150% strain), and 3.75 (150–250% strain) (Figure 7d). Furthermore, the hydrogel sensor exhibited exceptional response characteristics, including a rapid response and recovery times (269 ms and 87 ms, respectively) (Figure 7e). These collective results confirmed that the PVA/AA3-SBMA hydrogel displayed high sensitivity and stable response, establishing it as an ideal candidate for flexible sensing applications.

**Figure 8:**
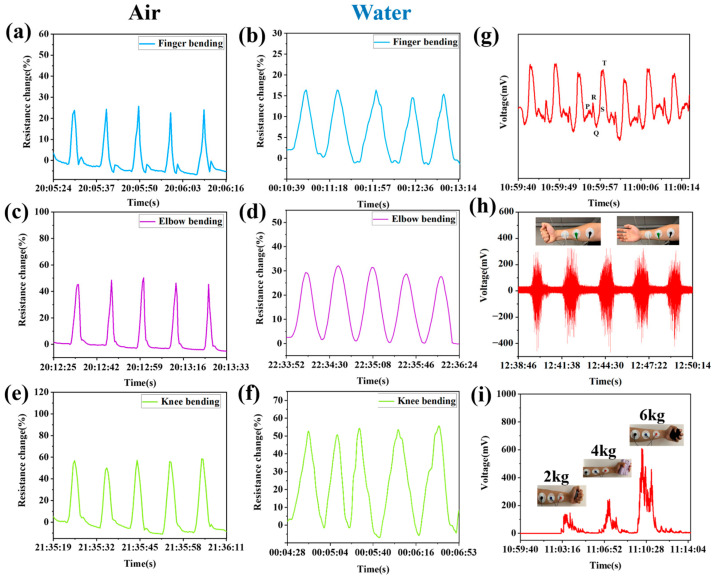
Human motion, ECG, and EMG detection of the PVA/AA-SBMA hydrogel. Resistance changes in finger bending: (**a**) in air and (**b**) in water. Resistance changes in arm bending: (**c**) in air and (**d**) in water. Resistance changes in knee bending: (**e**) in air and (**f**) in water. Human physiological signal detection: (**g**) ECG signals, (**h**) EMG signals of arm muscle tension, and (**i**) EMG signals under different strengths.

The PVA/AA3-SBMA hydrogel sensor was also successfully employed for real-time human motion detection across from both air and aquatic environments (Figure 8 and Movie S4). When hydrogel attached to various locations including finger, elbow, and knee, it demonstrated different and reliable electrical signal variation. The relative electrical signal change increased with the deformation amplitude increase in the hydrogel. Comparative analysis disclosed distinct environment-dependent response characteristics, where hydrogel yielded enhanced resistance change in air compared to water environments for finger and elbow bending. However, knee bending signals almost remained their original level. The possible reasons are listed below. First, finger and elbow joints are relatively small, and the muscles that drive them are relatively weak. When bending in water, the huge water fluid resistance will significantly hinder the range and speed of joint movement. Even if the tester subjectively tries to bend the “same range”, the actual bending angle and angular velocity achieved are likely to be lower than in air. The quadriceps muscles, which drive the knee flexion and extension, are among the strongest muscle groups in the human body. Compared to finger and elbow bending, the knee has enough strength to overcome fluid resistance in water and achieve movements closer to that in air [47,48]. Second, the output signal of resistance change amplitude for most hydrogel strain sensors directly depends on the applied strain/degree of deformation. For finger and elbow bending, the actual strain that occurs underwater is reduced, which directly leads to a decrease in the signal amplitude. For knee bending, the strain/degree of deformation actually applied to the hydrogel sensor is close to that in air. Therefore, the original signal amplitude generated by the hydrogel sensor is also maintained [49]. In addition, hydrogel demonstrated different response times and recovery times between water and air for human activity sensing. There are two possible reasons resulted in different response times and recovery times for human activity sensing. On the one hand, the density and viscosity of water are much higher than those of air. When the hydrogel sensor needs to deform to respond to movement, the surrounding water molecules will produce huge fluid resistance on the hydrogel sensor, leading to longer response and recovery times for underwater sensing [50]. On the other hand, the tester complete movement with different frequency during sensing process, thereby resulting in different response and recovery times. The human activity sensing performance of hydrogel across different motion amplitudes and environmental conditions confirmed its desirable adaptability and sensitivity. Furthermore, the hydrogel sensor exhibited potential for human physiological signal monitoring. As demonstrated in Figure 8g, the hydrogel precisely captured electrocardiogram (ECG) signals with clearly identifiable P-Q-R-S-T waveforms, confirming its ability in real-time ECG signal monitoring—a critical capability for healthcare diagnostics and therapeutic applications. Subsequent electromyogram (EMG) signal recordings (Figure 8h) revealed distinct signal variations corresponding to arm muscles contraction, demonstrating remarkable sensitivity to electrical potentials of muscles. Notably, the amplitude of these EMG signals exhibited a direct correlation with the weight of objects held in hand (Figure 8i), providing evidence for the superior sensitivity and dynamic response characteristics of the hydrogel. These collective findings indicated that the PVA/AA-SBMA hydrogel sensor possessed significant potential for advanced biotronics application even in underwater environments.

## 4. Conclusions

This study presented an innovative anti-swelling conductive hydrogel platform (PVA/AA-SBMA) through the construction of a dual-network structure, comprising copolymerized zwitterionic poly(SBMA–AA) as the primary network and physically crosslinked PVA as the secondary network. The hydrogel exhibited remarkable anti-swelling characteristics, achieving a low equilibrium swelling ratio of 80% after 120 h of immersion in DI water. When employed as a flexible strain sensor, the hydrogel demonstrated superior performance including high sensitivity with a gauge factor of 3.75 (150–250% strain), coupled with rapid response featuring a 269 ms of response time and 87 ms of recovery time. In addition, the hydrogel sensor demonstrated excellent real-time human activity monitoring capability, accurately detecting physiological and movement signals. This breakthrough significantly expanded the prospects of hydrogels in human activity sensing.

## Figures and Tables

**Table 1:** Content of each component in the hydrogel.

Designation	PVA	AA	SBMA
PVA/SBMA	1.0 g	/	1.2 g
PVA/AA1-SBMA	1.0 g	2.0 mL	1.2 g
PVA/AA2-SBMA	1.0 g	3.0 mL	1.2 g
PVA/AA3-SBMA	1.0 g	4.0 mL	1.2 g

## Data Availability

The original contributions presented in this study are included in the article and Supplementary Material. Further inquiries can be directed to the corresponding author.

## Supplementary Materials

The following supporting information can be downloaded at: https://www.mdpi.com/article/10.3390/polym17162230/s1, Figure S1: Electrical conductivity of hydrogels before swelling and after swelling for 24 h; Figure S2: Mechanical tensile performance demonstration of hydrogels after swelling for 24 h; Movie S1: Mechanical tensile performance of hydrogel; Movie S2: Mechanical compressive performance of hydrogel; Movie S3: Strain sensing property of hydrogel; Movie S4: Human motion sensing property of hydrogel.
