# Supplementary material for: Anti-Swelling Dual-Network Zwitterionic Conductive Hydrogels for Flexible Human Activity Sensing
Source: Polymers (Basel). 2025 Aug 16;17(16):2230. doi: 10.3390/polym17162230 (PMC12389484; doi:10.3390/polym17162230)
Supplement: Supplementary file 1 [file polymers-17-02230-s001.zip › polymers-3733723-supplementary.pdf]

**Supporting information**

# Anti-Swelling Dual-Network Zwitterionic Conductive Hydrogels for Flexible Human Activity Sensing

Zexing Deng [a, 1], Litong Shen [a, 1], Qiwei Cheng [a], Ying Li [a], Qianqian Liu [a], Xin Zhao [b, *]

[a] *College of Materials Science and Engineering, Xi'an University of Science and Technology, Xi'an, 710054, China*

[b] *State Key Laboratory for Mechanical Behavior of Materials, Xi'an Jiaotong University, Xi'an, 710049, China*

[1] Zexing Deng and Litong Shen contributed equally to this work.

Correspondence: Xin Zhao, Email: zhaoxinbio@mail.xjtu.edu.cn

## Results

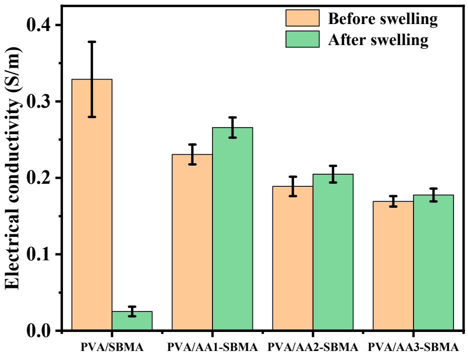


**Figure S1.** Electrical conductivity of hydrogels before swelling and after swelling for 24 h.

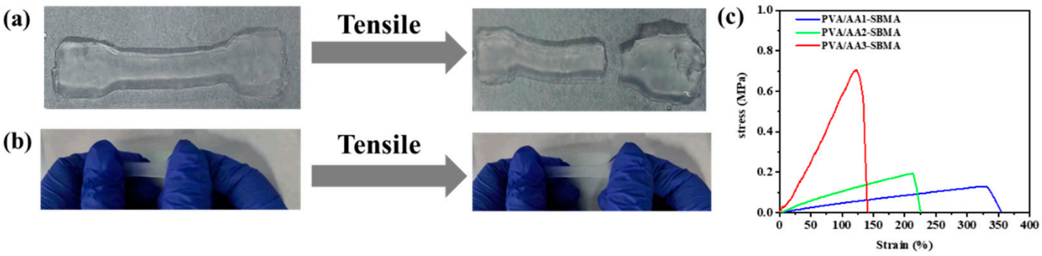


**Figure S2.** Mechanical tensile performance demonstration of hydrogels after swelling for 24 h. (a) PVA/SBMA hydrogel fractured under minor tensile force. (b) PVA/AA3-SBMA hydrogel without fracture under proper tensile force. (c) Mechanical tensile curves of PVA/AA-SBMA hydrogels.

**Movie S1.** Mechanical tensile performance of hydrogel.

**Movie S2.** Mechanical compressive performance of hydrogel.

**Movie S3.** Strain sensing property of hydrogel.

**Movie S4.** Human motion sensing property of hydrogel.
